# Erythropoiesis stimulating agents and reno-protection: a meta-analysis

**DOI:** 10.1186/s12882-017-0438-4

**Published:** 2017-01-11

**Authors:** Steve Elliott, Dianne Tomita, Zoltan Endre

**Affiliations:** 1 Amgen Inc, One Amgen Center, Newbury Park, Thousand Oaks, CA 91320 USA; 2 Department of Nephrology, Prince of Wales Hospital and Clinical School, University of New South Wales, Sydney, NSW 2031 Australia

**Keywords:** AKI (acute kidney injury), Anemia, Clinical trial, EPO, Erythropoietin, ESA, Meta-analysis, Progression of CKD, Reno-protection, Tissue protection, Transplant

## Abstract

**Background:**

Erythropoiesis stimulating agents (ESAs) were proposed to enhance survival of renal tissues through direct effects via activation of EPO receptors on renal cells resulting in reduced cell apoptosis, or indirect effects via increased oxygen delivery due to increased numbers of Hb containing red blood cells. Thus through several mechanisms there may be benefit of ESA administration on kidney disease progression and kidney function in renal patients. However conflicting ESA reno-protection outcomes have been reported in both pre-clinical animal studies and human clinical trials. To better understand the potential beneficial effects of ESAs on renal-patients, meta-analyses of clinical trials is needed.

**Methods:**

Literature searches and manual searches of references lists from published studies were performed. Controlled trials that included ESA treatment on renal patients with relevant renal endpoints were selected.

**Results:**

Thirty two ESA controlled trials in 3 categories of intervention were identified. These included 7 trials with patients who had a high likelihood of AKI, 7 trials with kidney transplant patients and 18 anemia correction trials with chronic kidney disease (predialysis) patients. There was a trend toward improvement in renal outcomes in the ESA treated arm of AKI and transplant trials, but none reached statistical significance. In 12 of the anemia correction trials, meta-analyses showed no difference in renal outcomes with the anemia correction but both arms received some ESA treatment making it difficult to assess effects of ESA treatment alone. However, in 6 trials the low Hb arm received no ESAs and meta-analysis also showed no difference in renal outcomes, consistent with no benefit of ESA/ Hb increase.

**Conclusions:**

Most ESA trials were small with modest event rates. While trends tended to favor the ESA treatment arm, these meta-analyses showed no reduction of incidence of AKI, no reduction in DGF or improvement in 1-year graft survival after renal transplantation and no significant delay in progression of CKD. These results do not support significant clinical reno-protection by ESAs.

## Background

Erythropoietin (EPO) is a circulating hormone produced by the kidney, that stimulates erythropoiesis by binding and activating the EPO receptors (EPOR) on erythroid progenitor cells [1]. Subjects with chronic kidney disease (CKD) often develop anemia because of decreased production of EPO resulting in insufficient erythropoiesis. The cloning of the EPO gene allowed treatment of anemia in CKD patients by stimulating erythropoiesis with rHuEpo or other erythropoiesis stimulating agents (ESAs) [2].

Chronic anemia can result in organ damage affecting the cardiovascular system, kidneys, and the central nervous system [3–6] thus anemia correction might improve outcomes. In addition, EPOR was reported in nonhematopoietic tissues including renal cells [1], with some preclinical data suggesting that ESAs may be reno-protective due activation of EPOR resulting in anti-apoptotic effects [7, 8]. Some data suggest ESAs are reno-protective through an EpoR:CD131 complex and that EPO derivatives lacking erythropoietic activity are still reno-protective [9]. Other data conflicts with both hypotheses [1, 10]. However, the possibility ESAs might mitigate the serious consequences of renal ischemia through direct (anti-apoptosis of renal cells) or indirect effects (increased oxygen delivery with increased Hb) resulted in clinical trials to assess the potential benefit of ESA treatment in humans with renal diseases, and analysis of the results of those trials is warranted.

Clinical interventions to see if there is a relationship between ESAs and renal outcomes included short-term prophylactic ESA treatment where there was a high likelihood of acute kidney injury (AKI), e.g., patients undergoing coronary artery bypass grafting (CABG) surgery. In another modality, ESA treatment at the time of surgery might mitigate the ischemic damage and delayed graft function (DGF) that occurs during the perioperative period following kidney transplant. DGF increases the risk of acute rejection, impaired graft function, and reduces long term patient and graft survival. In a third modality, treatment of CKD patients to correct anemia associated with renal failure presumes that ESA treatment might delay or prevent renal disease progression through direct anti-apoptotic effects on renal cells or indirect effects of anemia correction, eg improved oxygen delivery.

Most of the trials examining the effect of ESAs on renal patients were small, outcomes were not robust or they varied across studies. Therefore, results from individual trials were inconclusive, but meta-analyses of results from those clinical trials may allow more definitive conclusions. We reasoned further that meta-analysis of multiple modalities would add additional value. The three modalities above were selected for meta-analysis because they examined direct and/or indirect effects of ESAs on renal disease progression or renal function. We report here that meta-analyses show no significant beneficial effects in any of the modalities, suggesting that ESAs have little reno-protective benefits, at least with the patient populations examined and clinical designs employed.

## Methods

We wished to assess the effect of ESAs on kidneys by analyzing data from human clinical trials where ESAs might mitigate effects of ischemia or disease progression. This necessitated comprehensive searches and identification and analysis of controlled trials with renal patients where ESAs were used to protect kidneys from ischemia or to slow renal disease progression. All trials that had relevant renal endpoints were selected and analyzed, and data was extracted from those that might test the hypothesis.

### Search strategy

Literature searches were performed using OVIDSP (Wolters Kluwer companies) to access MEDLINE and other databases including Current contents, Embase and BIOSYS previews, using search terms for ESAs (EPO, erythropoietin, rHuEpo, rEpo, epoetin, darbepoetin) in combination with anemia terms (anemia, Hb, hemoglobin, hct, hematocrit), kidney or kidney injury (renal, kidney, transplant, CKD, chronic kidney disease, delayed graft function, DGF, acute kidney injury, and AKI), and terms describing possible beneficial outcome (protect, protection, reno-protection). Searches of the Clinicaltrials.gov and the Cochran database websites were performed using ESA terms combined with anemia, renal, kidney and transplant, to further identify potential papers of interest. A manual search of the reference lists in papers, review articles and other meta-analyses identified additional papers.

### Trial selection/inclusion criteria

Papers considered for inclusion described human clinical data with ESA treatment and renal endpoints. Papers were rejected if they were not controlled trials, were case reports, described only preclinical data, or lacked the relevant renal endpoints. Papers with ESA treatment of renal patients on dialysis were omitted because renal disease progression was not applicable. The final list included controlled clinical trials that utilized ESAs in transplantation, AKI, and for anemia correction in predialysis CKD patients.

### Data extraction

The data was recovered by SE and reviewed by ZE. Recovered data included the study characteristics, study location, length of study, ESA treatment, nature of the comparator arm, number of subjects in each arm, time intervals and definitions of renal endpoints. Results were grouped according to study type (patients presenting with or at risk of AKI, studies with kidney transplant patients, and CKD patients undergoing anemia correction). For trials involving AKI, data collected for meta-analysis was the number of patients with AKI and number of patients with renal recovery following AKI. Other endpoints recovered from those trials were any creatinine-based or enzymatic markers that were measures of renal function or renal injury. With kidney transplant studies the measures recovered for meta-analysis were incidence of DGF within the first week post-surgery and graft loss/survival over a 1 year period. Other data collected were any creatinine-based data, incidence of proteinuria, and enzymatic-based markers of renal injury. The meta-analysis endpoint in anemia correction trials was incidence of progression to renal replacement therapy (RRT; progression to dialysis or kidney transplant) at any time during the study. Other data recovered were, estimated glomerular filtration rate (eGFR), serum creatinine (sCr), and their rate of change over time, and incidence of proteinuria. All the trial information and secondary measures are summarized in Tables 2, 3 and 4. The data used in meta-analysis are shown in Figs. 3, 4, 5 and 6.

Data extracted to assess trial quality (bias) included randomization, concealment of allocation, masking of patients and clinicians, documentation of dropouts and withdrawals, and whether analysis was by intention-to-treat.

### Statistical analysis

Data were summarized using Comprehensive Meta-Analysis Software (V2) (Biostat, Inc., Englewood, NJ, USA). A random-effects model was used because it assumes treatment effects are not identical in all studies. However, results of analyses using a fixed-effects model, which assumes that the treatment effect is the same in each study and that differences in results are due only to chance, are also provided when the I^2^ statistic was not equal to zero. Risk ratios (RR) and 95% confidence intervals were calculated to compare results for patients treated with ESA with the control group. Heterogeneity or inconsistency across studies was assessed using Cochrane’s Q (*p*-value) and the I^2^ statistic. The *p*-value for the z-test comparing treatment groups was also determined.

## Results

### Description of searches and study selection criteria

**Fig. 1:**
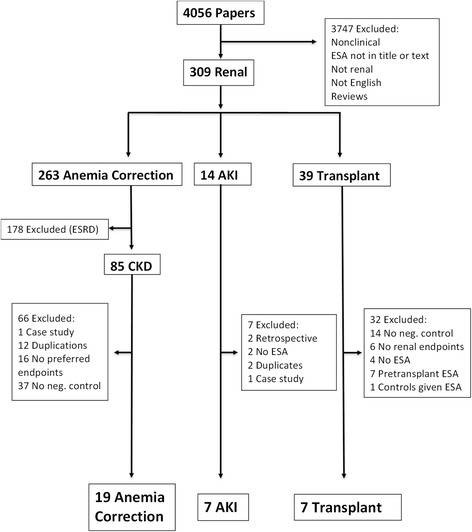
Flow chart of study selection

The titles of papers from the searches were reviewed, and abstracts examined. Papers with potential relevance to ESAs, human clinical trials and tissue protection were recovered. This process resulted in 4056 papers. The selection and rejection process for these papers is shown in Fig. 1. Papers describing non-human studies, were reviews, were not clinical trials, lacked renal endpoints, were not in English, did not include a term for anemia, Hb or an ESA in the paper, or they did not otherwise fulfill the inclusion criteria were excluded. The resulting 309 papers described clinical trials with ESA-treated subjects that fell into 3 categories, at risk or presenting with AKI, ESA-treated kidney transplant patients and patients undergoing anemia correction with ESAs. Papers describing trials on dialysis patients, trials lacking a control group, trials that did not use ESAs, or were case studies, were omitted. Choukroun 2012 [11] was an anemia correction trial on renal transplant patients and not CKD patients so it was omitted. In 3 trials, ESAs were given prior to renal transplant [12–15] and omitted because there could be no direct effect of ESA on the ischemic transplanted kidney. Duplications were identified; Oh 2012 [16] was a reanalysis of Song 2009 [17] and Revicki 1995 [18] was a follow-up of Roth 1994 [19]. The Park (2005) [20] and Olweny (2012) [21] trials were excluded from meta-analysis because they were retrospective trials without AKI endpoints. 33 papers published between 1989 and 2015 remained, and their characteristics and extracted data are summarized in Tables 2, 3 and 4. Measures of renal function (sCr, eGFR, and enzymatic) varied, (methods and times), or were not reported in many papers. Therefore, we chose not to perform meta-analyses using those markers but instead summarize available data in the tables. Meta-analyses (Forrest plots) using the selected hard endpoints, are shown in Figs. 3, 4, 5 and 6.

### Risk of bias assessment

**Fig. 2:**
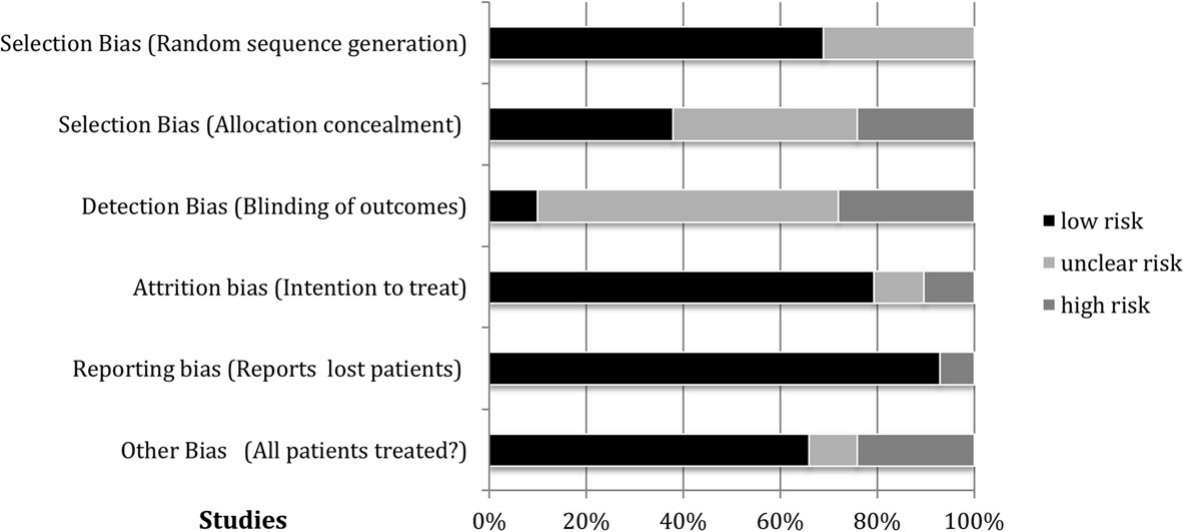
Risk of bias graph

Trial quality (potential bias) was evaluated utilizing Jadad [22] and Cochrane recommendations. With the exception of Kamar 2010 [23] (which was a observational trial) all the trials used in meta-analysis were RCTs. Risk of bias assessment is shown in Table 1 and Fig. 2. Most trials provided an ITT analysis with reporting of lost patients. The trials also had adequate methods to randomly distribute subjects into intervention vs control groups. Blinding of subject distribution and blinding of outcome to assessors was inadequate in most trials, particularly the anemia correction trials. However, the hard renal endpoints used in these meta-analyses are strengths. Most AKI and transplant trials were double-blinded with few dropouts, while the anemia correction trials were mostly open-label with variable numbers of dropouts. Overall, the trials had a risk of bias that was considered acceptable and thus results from meta-analysis would be informative.

**Table 1:** Assessment of Risk of Bias of Randomized Controlled Trials

Reference	Trial features	Randomized sequence	Allocation concealment	Blinding of outcome assessors	ITT analysis	Reports on Lost patients	All patients treated in assigned group
Dardashti 2014 [24]	AKI: DB, SS	Low risk: patients were randomly allocated.	Low risk: sequentially numbered, sealed, & opaque envelopes. Independent nurses prepared the study drug & syringes were delivered blinded	High risk	High risk: 5 patients that received study drug were discontinued and excluded from analysis	Low risk: lost patients reported	Low risk: all patients treated
deSeigneux 2012 [76]	AKI: DB, SS	Low risk: a randomization code was generated by computer	Low risk: envelopes with allocation were prepared by the quality of care unit. A nurse opened the envelopes and prepared the syringes for injection. Investigators and patients were blinded to the treatment	High risk	Low risk: AKI data on all patients	Low risk: lost patients reported	Low risk: all patients treated
Endre 2010 [26]	AKI: DB, MS (2 centers)	Low risk: allocation by a predefined computer-generated randomization sequence	Low risk: concealment was by a pharmacist; pairs of identical syringes. Patients, all medical staff, & investigators were blinded to treatment	Low risk: Data Safety Monitoring Board with unmasking followed recording of the final AEs of the patient last enrolled	Low risk	Low risk: lost patients reported	Low risk: but 1 patient withdrew
Kim 2013 [27]	AKI: DB, SS	Low risk: computer-generated random code	Low risk: medications were prepared by a nurse who knew the patient’s group assignment but was not involved in the study	Unclear risk	Low risk: No dropouts	Low risk: lost patients reported	Low risk: all patients treated
Oh 2012 [16]	AKI: DB, SS	Low risk: A randomization code list with a block size of two was generated. Treatments were allocated to patients through the Internet in accordance with the predefined randomization list	Low risk: a research coordinator performed randomization and prepared the study drugs	Unclear risk	Low risk	Low risk: all patients completed the trial	Low risk: all patients completed the trial
Tasanarong 2013 [28]	AKI: DB, SS	Low risk: treatment assignment by blocked randomization. Sealed envelopes containing the allocation group were opened by nurses who did not participate in the study	Low risk: treatments were blindly given to the research coordinator. Patients and investigators were blinded to group assignment. Pairs of identical syringes containing either rHuEPO or saline were prepared	High risk	Low risk: No dropouts	Low risk: no dropouts	Low risk: no dropouts
Yoo 2011 [29]	AKI: OL(single blinded), SS	Low risk: patients were allocated by computer-generated random numbers	Unclear risk: medications were prepared and administered by a ward physician recognizing the patient’s group but not involved in the current study, the surgeon and anesthesiologist involved were blinded	Low risk: the surgeon and anesthesiologist involved in the study and patient management were blinded to the patients’ groups until the end of the study	Low risk: complete data sets from the 74 patients were analyzed without any missing data	Low risk: no dropouts	Low risk: complete data sets from the 74 patients were analyzed without any missing data
Aydin 2012 [31]	Transplant: DB, SS	Low risk: Patients were randomized by an independent hospital pharmacist. The randomization allocation sequence was generated by a random-number table	Low risk: patients, physicians, data managers and investigators were kept blinded throughout the study	Low risk: data managers and investigators were kept blinded throughout the study	Low risk: No dropouts	Low risk: No dropouts	Low risk: No dropouts
Coupes 2015 [30]	Transplant: DB, SS	Low risk: patients were randomly assigned by the trial pharmacy by computer	Low risk: all study participants and the study team were blinded to the trial drug	Unclear risk	Low risk: 1 patient withdrew but was included in the analysis	Low risk: lost patients reported	Low risk
Hafer 2012 [32]	Transplant: DB, SS	Unclear risk: randomization methodology not disclosed	Low risk: vials containing ESA and placebo had identical appearance	Unclear risk	Low risk for DGF. High risk for graft loss (3 patients died 1 in ESA group and 2 in placebo group)	Low risk: lost patients reported	High risk: 2 untreated patients (not included in analysis) and 3 patients died
Martinez 2010 [33]	Transplant: OL, MC	Unclear risk: randomization method not disclosed	High risk: comparator arm was untreated	Low risk: Blinded evaluation of end-points	Unclear risk: 1 died in ESA group	Low risk: lost patients reported	Low risk
Sureshkumar 2012 [34]	Transplant: DB, SS	Low risk: the hospital pharmacy created a schedule using random assignments to a series of patient study numbers	Low risk: ESA and placebo were both 1 ml syringes. The medications were administered in a double-blinded manner	Unclear risk	Low risk	Low risk: no dropouts	Low risk
Van Biesen 2005 [35]	Transplant: OL, SS	Unclear risk: randomization method not disclosed	High risk: open label	High risk	Unclear risk	High risk	Unclear risk
Van Loo 1996 [36]	Transplant: OL, SS	Unclear risk: randomization method not disclosed	High risk: open label	High risk	Low risk: no deaths or withdrawals	Low risk: no deaths or withdrawal	Low risk: no deaths or withdrawals
Abraham 1990 [38]	Anemia correction: DB then OL, Anemia correction: SS	Unclear risk: randomization method not disclosed	Unclear risk: unspecified	High risk	Low risk: no dropouts	Low risk: no dropouts	Low risk
Clyne 1992 [39]	Anemia correction: OL, 2 center	Unclear risk	High risk	High risk	Low risk: for RRT	Low risk: lost patients reported	Low risk
Kleinman 1989 [40]	Anemia correction: DB, MC	Unclear risk: randomization method not specified	Unclear risk: unspecified	High risk	Unclear risk: no dropouts reported	Unclear risk: no dropouts reported	Low risk
Kuriyama 1997 [41]	Anemia correction: OL, SS	Unclear risk	High risk	High risk	Low risk	Low risk: lost patients reported	Low risk
Lim 1989 [42]	Anemia correction: DB, SS	Low risk: randomization by third party	Unclear risk	Unclear risk	High risk	Low risk: lost patients reported	Low risk
Lim 1990 [43]	Anemia correction: OL, SS	Unclear risk	High risk	High risk	Low risk: no dropouts	Low risk: no dropouts	Low risk
Revicki 1995 [18]	Anemia correction: OL, MC	High risk	High risk	High risk	Low risk: for RRT endpoint	Low risk: lost patients reported	Unclear risk
Cianciaruso 2008 [45]	Anemia correction: OL, MC	Low risk: randomization by computer at a separate site	Low risk: allocation was concealed from investigators, sequences were sequentially numbered in opaque envelopes opened in sequence	High risk	Low risk	Low risk: lost patient reports	High risk: 1 patient in the treatment group did not receive ESA, study terminated early
Gouva 2004 [47]	Anemia correction: OL, MC	Low risk: computer generated sequence	Unclear risk	High risk	Low risk	Low risk: lost patients reported	High risk: study prematurely terminated
Levin 2005 [48]	Anemia correction: OL, MC	Low risk: computer generated sequence	Low risk: allocation was in sealed sequentially numbered opaque envelopes. Designated personnel opened the next number in sequence	High risk	Low risk	Low risk: lost patient reports	High risk: only 77/85 in the high Hb group received ESA
MacDougall 2007 [49]	Anemia correction: OL, MC	Low risk: randomized using central randomization procedures (ClinPhone)	Unclear risk	High risk	Low risk	Low risk: lost patients reported	High risk: patients in the high Hb group received ESA on day 1 but study was prematurely terminated
Pfeffer 2009 [50]	Anemia correction: DB, MC	Low risk: DB, and patients were randomly assigned with the use of a computer-generated, permuted-block design	Unclear risk	High risk	High risk: 9 patients were excluded prior to unblinding	Low risk: lost patient reports	High risk: 93.9% of the patients in the darbepoetin alfa group were receiving the assigned treatment at 6 months”
Ritz 2007 [51]	Anemia correction: OL, MC	Low risk: randomization was performed centrally into treatment groups by using a block-size randomization procedure stratified by country	Unclear risk	High risk	Low risk	Low risk: lost patient reports	Unclear risk: patients in group 1 were started immediately ESA but 3 patients withdrew
Roger 2004 [52]	Anemia correction: OL, MC	Low risk: patients were randomized according to computer-generated stratification tables	Low risk: order concealment was maintained until the intervention was assigned	High risk	Low risk	Low risk: lost patient reports	Low risk
Rossert 2006 [53]	Anemia correction: OL, MC	Low risk: patients were randomized according to computer-generated stratification schedule	Unclear risk	High risk	Low risk	Low risk: lost patient reports	High risk: study was terminated prematurely. Many subjects did not enter maintenance or withdrew
Villar 2011 [55]	Anemia correction: OL, MC	Low risk: block-size randomization was used	Unclear risk	High risk	Low risk	Low risk: lost patients reported	Unclear risk: most patients likely received ESA but 6 patients died or withdrew
Akizawa 2011 [44]	Anemia correction: OL, MC	Low risk: patients were assigned by a computer according to a minimization method	Unclear risk	High risk	Low risk	Low risk: lost patients reported	High risk: after 1 administration, 43 withdrew.
Drueke 2006 [46]	Anemia correction: OL, MC	Low risk: randomization was performed centrally with the use of a dynamic randomization method	Unclear risk	High risk	Low risk	Low risk: lost patients reported	High risk: 75 in the high Hb group withdrew
Singh 2006 [54]	Anemia correction: OL, MC	Low risk: patients were assigned by computer-generated per-muted-block randomization	Unclear risk	High risk	Low risk	Low risk: lost patients reported	High risk: study was terminated early at the second interim analysis because power to demonstrate benefit was less than 5%, and there was a high withdrawal rate

*RCT-randomized controlled trial, *DB* Double blind, *OL* Open label, *MC* Multicenter, *SC* Single center

### Outcomes and meta-analysis

#### AKI trials

Nine trials were identified [16, 20, 21, 24–29] that assessed whether ESAs might reduce the risk of AKI (Table 2). In 8 trials the subjects underwent cardiac surgery (coronary artery grafting, or valvular heart surgery involving cardiopulmonary bypass) and in 1 trial the subjects underwent partial nephrectomy. The combined number of subjects was 1020; 490 in the ESA groups and 530 in the control groups. The trial sizes ranged from 71 to 187 subjects. The number of ESA administrations were small (1 or 2) so there were little/no changes in Hb (Table 2).

**Table 2:** AKI studies

Reference	Study Location	Patient Population	ESA	Control	Subjects (Total and # in groups)	Renal Injury (AKI) Definition	Other Outcomes
Dardashti 2014 [24]	Sweden (Skåne University Hospital, Lund)	Patients scheduled for CABG with preexisting renal impairment	Epoetin zeta (400 IU/kg; Retacrit®) administered preoperative	Equivalent volume of saline	*N* = 70: ESA(35), control(35)	RIFLE on d3 based on eGFR using the Modification of Diet in Renal Disease formula	No difference in Hb, transfusions, relative cystatin C, NGAL, creatinine, urea, or eGFR)
deSeigneux 2012 [76]	Switzerland (University Hospital, Geneva)	Patients admitted to the ICU for cardiac surgery	ESA Group 1 (20,000 IU; epoetin α), group 2 (40,000 UI epoetin α) & group 3 (control) 1 to 4 h post-surgery	Isotonic sodium chloride	*N* = 80: ESA group 1(20), ESA group 2(20), control(40)	AKIN from ICU admission to the following wk	No difference in Hb, creatinine, cystatin c, or urinary NGAL levels
Endre 2010 [26]	New Zealand (Christchurch or Dunedin Hospital)	Patients admitted to the ICU or high-risk patients scheduled for cardiothoracic surgery with CPB	ESA (500 U/kg (iv) to a maximum of 50,000 U), within 6 h of increased GGT AP and a second dose 2 h later	Equivalent volume of normal saline	*N* = 163: ESA(84), control(78)	AKIN classification in 7 days	No difference in any creatinine-based variables
Kim 2013 [27]	Korea (Yonsei University Health System, Seoul)	Patients with preoperative risk factors for AKI who were scheduled for complex valvular heart operations	Epoetin α (300 IU/kg (iv); Epocain) after anesthetic induction	Equivalent volume of normal saline.	*N* = 98: ESA(49), control(49)	An increase in serum creatinine >0.3 mg/dl or >50% from baseline:	No differences in Hb, sCr, eGFR, creatinine clearance, cystatin C or serum NGAL
Olweny 2012 [21]	USA (UT Southwestern, Houston, Texas)	Patients who underwent laparoscopic partial nephrectomy	Epoetin α (500 IU/kg (iv) Procrit) 30 min prior to LPN	No ESA	*N* = 106: ESA(52), control(54).	NA	No difference in eGFR
Oh 2012 [16]	Korea, National University Bundang Hospital, Seoul	Patients scheduled for elective CABG	Epoetin β (300 U/kg Recormon) before CABG	Saline	*N* = 71: ESA(36,) control(35).	SCr ≥ 0.3 mg/dL from baseline, ≥50% increase in the sCr concentration in the first 72 h after CABG, or <0.5 mL/kg per hour of oliguria for more than six hr	sCr was not different from baseline in the ESA group, but was higher in the placebo group.
Park 2005 [20]	USA (surgical ICU), cardiothoracic ICU, or medical ICU at Barnes-Jewish Hospital, St Louis, Missouri)	Patients scheduled for elective CABG	ESA (112 U/kg/week average) within the first 14 days of RRT initiation	No ESA	*N* = 187; ESA(71), control(116)	NA	No difference in transfusions. sCr at 2 weeks favored the ESA arm but did not reach statistical significance (*p* = 0.054). No difference in renal recovery or renal survival
Tasanarong 2013 [28]	Thailand (Thammasat Chalerm Prakiat Hospital)	Patients scheduled for elective CABG using CPB	epoetin β (200 U/kg; Recormon) 3 d before CABG and 100 U/kg at the operation time.	Same volume & schedule of 0.9% saline	*N* = 100: ESA(50), control(50)	≥0.3 mg/dl or ≥50% increase in sCr from baseline within the first 48 h post-operation according to the KDIGO 2012 criteria.	No difference in Hb. sCr increase and eGFR decrease was lower in the ESA group. Mean urine NGAL group was lower in the ESA group 2 h & 18 h.
Yoo 2011 [29]	Korea (Yonsei University Health System, Seoul)	Patients scheduled for valvular heart surgery (VHS) with preoperative anemia	Epoetin α (500 IU/kg (iv); Epocain and 200 mg iron sucrose (iv)) 16-24 h pre-surgery	Equivalent volume of normal saline	*N* = 74: ESA(37), control(37)	Increased sCr of 0.3 mg/dl, or 50–200% from baseline, using modified RIFLE classification within 48 h after surgery	Reduced transfusions. No difference in mortality

The endpoint tested in the meta-analysis was the number of patients that developed AKI within 2–7 days (>50% increase serum creatinine, or >0.3 mg/dl increase, AKIN definition). Four of the trials were performed by overlapping members of the same study groups [16, 17, 27, 29]. Song (2009) and Oh (2012) analyzed the same 71 patients and patient data, but used different definitions of AKI. They increased the duration of observation to 72 instead of 48 h, and therefore had different numbers of patients that progressed to AKI. We used the determinations from Oh (2012) because it is more recent and the definition used is more complete (AKIN).

**Fig. 3:**
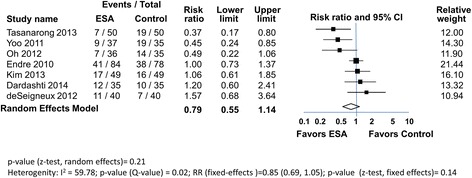
ESAs and incidence of AKI in patients at risk for AKI

Overall 107 of 367 (29%) of the subjects developed AKI in the ESA groups, with 133 of 357 (37%) in the control groups (Fig. 3). The RR slightly favored the ESA arm, but it did not reach statistical significance using either the random effects (0.79 [0.55, 1.14]), or fixed effects models (0.85 [0.69, 1.05]). Heterogeneity was high (I^2^ = 60%), 3 trials showed benefit in the ESA arm, while the other 4 were neutral, or favored the control arm. This heterogeneity is further apparent when other renal endpoints were examined (Table 2). In 1 trial [20] there was no difference in renal recovery, in 4 trials there was no difference in creatinine-based markers. However, in a 5th mixed results were reported. In a 6th creatinine markers favored slightly (*p* = 0.054) the ESA group and in the 7^th^, creatinine-based markers favored the ESA group. In 3 trials there was no difference in eGFR between groups, while in another trial, eGFR was improved in the ESA arm. Overall the secondary outcome analyses using non-creatinine-based renal biomarkers did not demonstrated significant reno-protection by ESAs. In 3 trials urine or plasma NGAL or serum cystatin C) were the same in both groups; in the 4th, urinary NGAL was lower in the ESA arm, although the significance of this difference is uncertain.

#### Renal transplant trials

Reinstitution of blood flow in cadaveric or live donor kidneys activates a sequence of events that results in renal injury, which may result in the development of DGF. DGF can translate into a decrease in long-term graft survival. In most ESA trials in transplant patients [14, 23, 30–36], DGF was defined as a requirement for dialysis within 7 days of the transplant [37]. In trials where multiple definitions were presented, data according to this definition was used. However, in some papers the definition of DGF was not disclosed, or an alternate measure was used (Table 3). The trial sizes were small to moderate in size (29–181 subjects). Like AKI trials, the number of ESA administrations were limited with little/no change in Hb.

**Table 3:** Kidney transplant studies

Reference	Study Location	ESA	Control	Subjects (Total and # in groups)	DGF definition	Other Outcomes
Aydin 2012 [31]	Netherlands (Leiden University Medical Center)	Epoetin β (33,000 IU) on 3 consecutive d, starting 3–4 h before transplantation & 24 & 48 h post-reperfusion.	Saline solution (0.9%)	*N* = 92: ESA(45), control(47)	Need for dialysis in the first wk or if sCr increased, remained unchanged or decreased by less than 10% per d during 3 consecutive d for more than 1 week	No significant differences in Hb, endogenous creatinine clearance or proteinuria
Coupes 2015 [30]	United Kingdom (Manchester Royal Infirmary)	Epoetin β (100,000 U; 33,000 intraoperative and 33,000 at 24 and 48 h).	Placebo (not disclosed)	*N* = 39: ESA(19), control (20)	Need for dialysis in first 7 days post-transplant	No difference in Hb or number of transfusions. No significant difference in sCr or eGFR at any time point to 90 day, No difference in acute rejection episodes, or biomarkers (NGAL, KIM-1 or IL-18)
Hafer 2012 [32]	Germany (Hannover Medical School)	Epoetin α (40,000 U (iv); Eprex) immediately before reperfusion and d3 and d7 after transplantation	Placebo (not disclosed) same volume and appearance	*N* = 88: ESA (44), control (44)	Urine output of less than 500 ml in the first 24 h after transplantation and/or need of dialysis because of graft dysfunction within the first wk after transplantation	Higher Hb at 2 and 4 but not 6 weeks. No significant difference in transfusions, eGFR 6 weeks or 12 months. No significant differences 6 weeks and 6 months post-transplant in histological indices.
Kamar 2010 [23]	France (Department of Nephrology, Dialysis and Organ Transplantation, CHU Rangueil, Toulouse)	Epoetin α or epoetin β (250 IU/kg/week) on d5 post-transplant, unless Hb level was above 12 g/dl for women and 13 g/dl for men. Cumulative ESA dose (D30) was 727 ± 499 IU/kg.	No ESA during the first month post-transplantation unless Hb dropped to <8 g/dl)	*N* = 181: ESA (82), control (99)	NA	Reduced Hb in ESA arm. No difference in transfusions. sCr levels were similar in both groups at 3, 6 and 12 months post-transplantation
Martinez, 2010 [33]	France (13 centers)	Epoetin β (30.000 IU; Neorecormon) given before surgery and at 12 h, d7 and d14	No ESA during the first month post transplantation	*N* = 104: ESA (51), control (53)	The need for dialysis during the first wk after transplantation	Higher Hb in ESA arm at 1 month. No difference in transfusions. No difference in sCr at any time point. No difference in eGFR at 1 or 3 months
Sureshkumar 2012 [34]	Pennsylvania (USA) (Allegheny General Hospital, Pittsburgh, Pennsylvania)	Epoetin α (100,000 U (iv); Procrit) intraarterially immediately after reperfusion	Matched placebo (not disclosed)	*N* = 72: ESA (36), control (36)	The need for dialysis within the first wk of transplantation	No difference in Hb, sCr, eGFR or urinary biomarkers of AKI (NGAL or IL-18)
Van Biesen 2005 [35]	Belgium (University Hospital Ghent)	Epoetin β (100/IU/kg; Recormon) immediately after transplantation then thrice weekly to maintain Hb above 12 g/dL	No ESA	*N* = 26: ESA (14), control (12)	Not defined	Shorter time to target Hb in ESA arm. No difference in transfusions or sCr at 3 months
Van Loo 1996 [36]	Belgium (University Hospital, Gent, Belgium)	Epoetin β (within 1 week post transplant). Starting dose was 150 U/kg 3X/week (sc), for a maximum of 12 weeks to maintain Hct between 25% and 35%.	No ESA	*N* = 29, ESA (14), control (15)	T1/2 sCr (the time for sCr to reach 50% of the pre-transplantation value for more than 2.5 days)	Increased Hb and reduced transfusions in ESA arm. No difference in sCr at any time point.

**Fig. 4:**
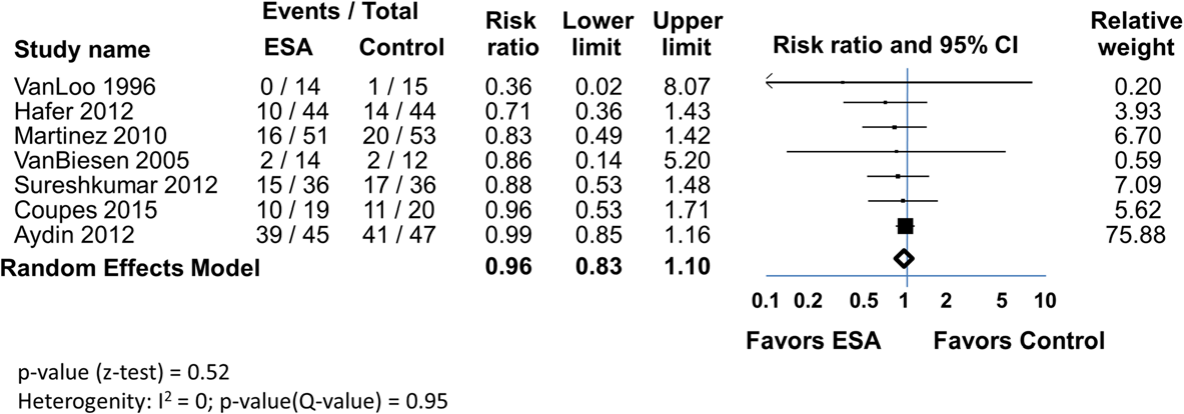
ESAs and DGF in patients undergoing kidney transplant

A meta-analysis with 450 subjects utilizing the DGF endpoint (7 trials), is shown in Fig. 4. DGF developed in 92 of 223 (41%) in the ESA arms and 106 of 227 (47%) in the control arms. The RR was neutral using random or fixed effects models (0.96 [0.83, 1.10]. Heterogeneity was low (I^2^ = 0%).

**Fig. 5:**
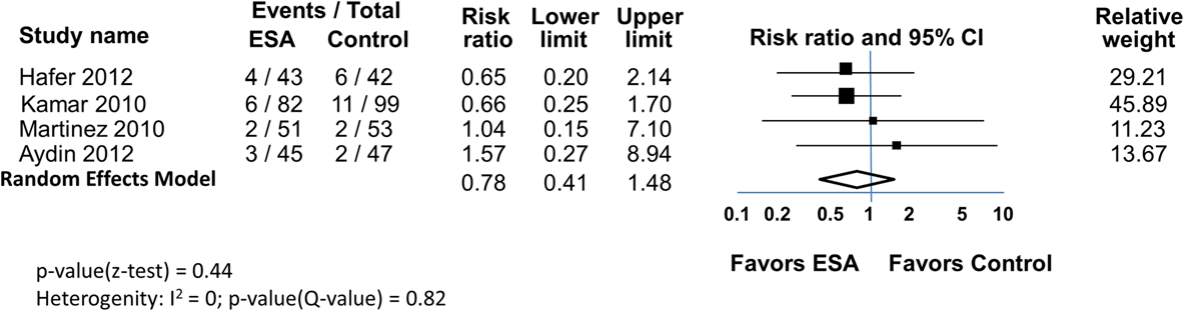
ESAs and graft loss in patients undergoing kidney transplant

Meta-analysis of long term graft loss over 1 year in four trials showed similar outcomes (Fig. 5). Fifteen of 221 subjects (6.8%) had graft loss in the ESA arms and 21 of 241 (8.7%) in the control arms. The RR (0.78 [0.41, 1.48]) slightly favored the ESA arm but did not reach statistical significance. Heterogeneity was low (I^2^ = 0%). Excluding the retrospective study [23] reduced the apparent benefit with 9/139 (6.5%) in the ESA arm and 10/142 (7.0%) having graft loss, and the RR was closer to neutral, but with a larger range (0.90 [0.37, 2.15]).

In the 7 trials, additional renal outcomes were reported that showed no differences between ESA and no-ESA groups (Table 3). These included creatinine-based endpoints (6 trials), eGFR (3 trials), proteinuria (1 trial), histological indices in graft biopsies at 6 weeks and 6 months post-transplant (1 trial), and low molecular weight urinary protein AKI biomarkers (NGAL and IL-18) (1 trial) [34].

#### Anemia correction trials

CKD patients are often anemic, and ESA treatment to increase and maintain Hb levels is long-term. Therefore, analysis of ESA anemia correction clinical trials is a potentially useful method to assess the effect of Hb increases, and oxygen delivery to renal tissues, on renal disease progression.

**Fig. 6:**
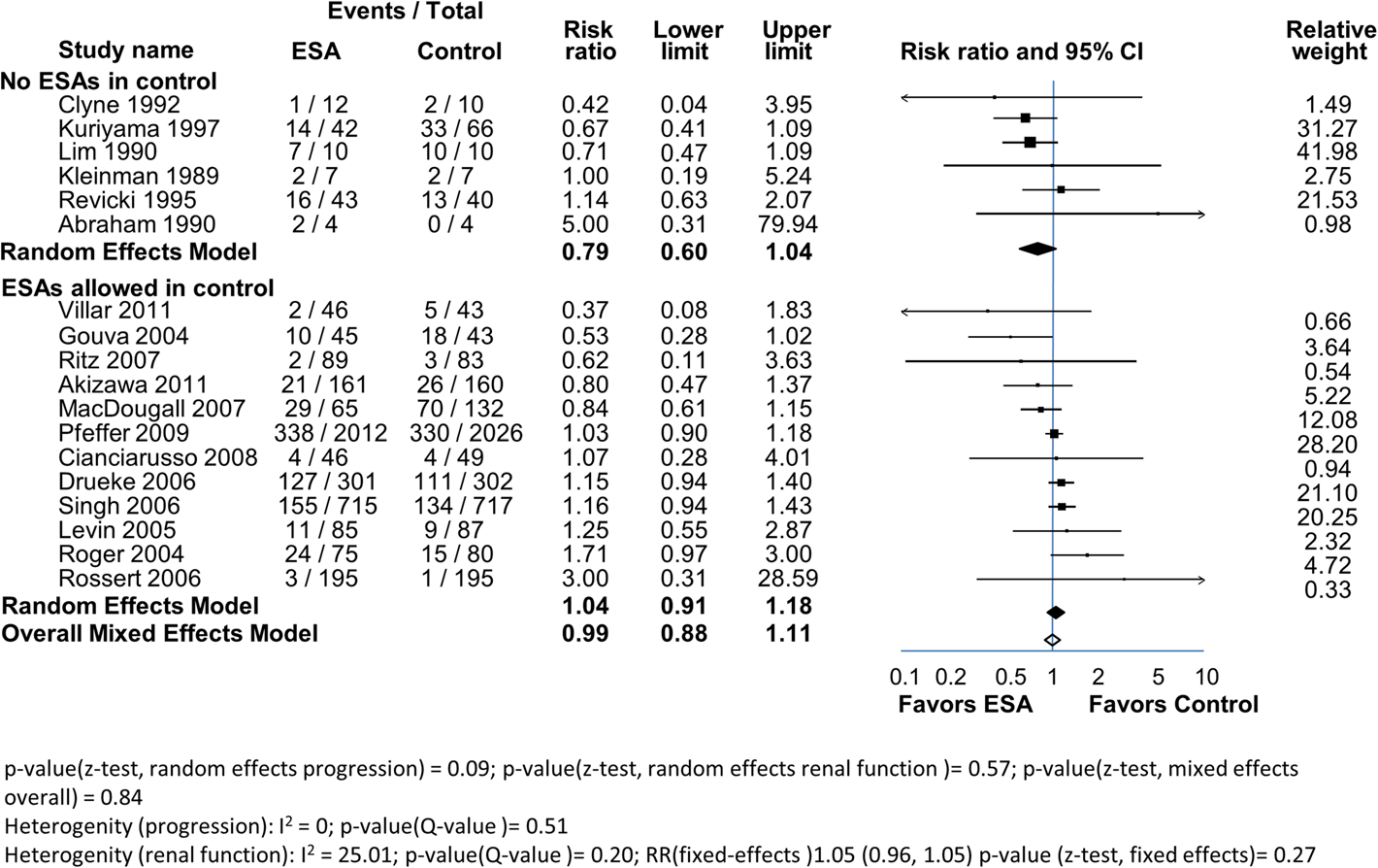
ESAs in anemic CKD patients. The 18 trials were divided into 2 groups. In 6 trials there was no ESAs administered in the control group. In 12 trials some patients in the control groups were given ESAs. The RR and range for each group (filled diamonds) and the overall RR (open diamond) are shown

In the 19 anemia correction trials identified, CKD patients were typically divided into 2 groups; those remaining at their starting Hb (control) and those where ESAs were used to target a higher Hb. ESAs in the 19 trials [18, 38–55] were typically given 1-3 times per week to raise and maintain target Hb levels (Table 4). The achieved Hb levels in most trials were 11–13.5 g/dL, with increases of 1–2.5 g/dL above the starting level. Trial duration ranged from 2 to 48 months. Many subjects in the lower Hb groups received ESAs, but at lower doses. In some trials, there was no ESA treatment of patients in the control groups. We performed meta-analysis on all trials and a separate meta-analysis of trials where subjects in the control groups did not receive ESAs (Fig. 6).

**Table 4:** Anemia correction studies

Reference	Study Location	ESA	Duration of Therapy	Comparator Arm	Subjects (Total and # in groups)	Starting vs Achieved Hb High(H) or low(L) Hb Group (g/dL)	Other Renal Outcomes
Abraham 1990 [38]	Hennepin County Medical Center Minneapolis Minn (USA)	Epoetin α (50–150 U/Kg 3X/w) to raise Hct to 37% vs 29%.	8–12 weeks to raise Hct then patients received ESA	Placebo (unspecified)	*N* = 8: ESA(4), control(4)	L: 9.3 vs 9.7 H: 10.7 vs 12.3	After 18 weeks there was no difference in the 1/sCr curves and no difference in protein excretion
Clyne 1992 [39]	Karolinska Hospital, Danderyd Hospital Stockholm (Sweden)	Epoetin β (300 U/kg) 1X/week to raise Hb from 8.6 to 11.7 g/dL	12 weeks	Placebo (unspecified)	*N* = 22: ESA(12), control(10)	L: 9.3 vs 9.4 H: 8.7 vs 11.3	No change in eGFR in either group. No significant difference in change in sCr
Kleinman 1989 [40]	Valley Presbyterian hospital, Van Nuys California (USA)	ESA (100 U/kg, 3x/week) to raise hct from 28 to 38–40%	12 weeks	Placebo (unspecified)	*N* = 14: ESA(7), control(7)	L: 9.4 vs 9.4 H: 9.4 vs 11.9	No difference in sCr or change in sCr
Kuriyama 1997 [41]	Saiseikai Central hospital, Tokyo Japan	Epoetin β (6000 U/week) to raise hct from 25.5 to 35.5%	36 weeks	No ESA	*N* = 108: ESA(42), control(66)	L: 9.3 vs 8.4 High Hb control 12.0 vs 10.7 H: 9.0 vs 11.8	Time to a doubling in sCr significantly slower in the ESA group.
Lim 1989 [42]	University of Iowa Hospitals’ Renal Clinic, Iowa (USA)	ESA (50, 100, or 150 U/kg 3X/week)	8 weeks	Placebo (unspecified)	*N* = 13: ESA(11), control(2)	L: 9,0 vs 12.7 H: 9.0 vs 8.0	No change in renal function over 2 months in ESA group
Lim 1990 [43]	University of Iowa Hospitals’ Renal Clinic, Iowa (USA)	Epoetin α 3X/week, later switched to 1X/week to raise Hct from 28 to 36%	11.8 ± 6.8 months (range 2.8-23.8)	No ESA	*N* = 20: ESA(10), control(10)	L: 11.0 vs 9.0 H: 9.3 vs 12.0	The rate of change in sCr was similar over 12 months
Revicki 1995 [18]	USA	Epoetin α (50 U/kg, 3X/week) then titrated to increase Hct from 27 to 35%.	48 weeks	No ESA	*N* = 83: ESA(43), control(40)	L: 8.9 vs 8.6 H: 8.9 vs 10.5	No difference in change in eGFR after 48 weeks, no difference in time to dialysis
Akizawa 2011 [44]	Japan	Darbepoetin alfa (30 ug 1X/week) to target Hb 11–13 g/dL.	48 weeks	rHuEpo (~4000 U/week) to maintain Hb at 9–11 g/dL. All received at least one dose of ESA	*N* = 321: High Hb (161), Low Hb (160)	L: 9.2 vs 10.1 H: 9.2 vs 11.9	No difference in 2 years decline in eGFR
Cianciaruso 2008 [45]	Italy	Epoetin α (2000 U 1x/week) to maintain Hb at 12–14 g/dL	12 months	No ESA unless Hb dropped below 9 g/dL. 2/49 received ESA	*N* = 95: High Hb (46), Low Hb (49)	L: 11.7 vs 11.4 H: 11.6 vs 12.4	No significant difference in eGFR or sCr
Drueke 2006 [46]	94 centers 22 countries	Epoetin β to raise Hb to a target of 13–15 g/dL. Median was 5000 U 1X/week	48 months	Hb targeted to >10.5 g/dL. ESA only if Hb dropped below 10.5 g/dL. 67% received ESA during the study. Median 2000 U 1X/week	*N* = 603: High Hb (301), Low Hb (302)	L: 11.6 vs 11.4 H: 11.6 vs 13.5	No significant difference in the last eGFR value before initiation of dialysis. Time to initiation of dialysis was shorter in the high Hb group at 18 months (*P* = 0.03).
Gouva 2004 [47]	Greece	Epoetin α (50 U/kg 1x/week) to raise Hb from 9–11.6 g/dL to a Hb target of 13 g/dL	Treatment time was a median of 22.5 months (range 16–24)	No ESA for a median of 12 months (range 7–19), then no ESA unless Hb dropped below 9 g/dL.	*N* = 88: High Hb(45), Low Hb(43)	L: 10.1 vs 10.3 H: 10.1 vs 12.9	No difference in sCr
Levin 2005 [48]	Canada	Epoetin α (2000 U 1X/week) to raise and maintain Hb at 12.0–14.0 g/dL	24 months	Low Hb (<11 g/dL), 16/74 received ESA	*N* = 172: High Hb(85) Low Hb(87)	L: 11.7 vs 11.4 H: 11.8 vs 12.8	No difference in creatinine clearance. Change in eGFR slower in the treatment group (not significant)
MacDougall 2007 [49]	United Kingdom	Epoetin α (1000 U 2X/week) to maintain Hb at 11.0 g/dl. Total was 190,000 U	3 years	No ESA until Hb dropped below 9 g/dL (55/132 received ESA; total 152,000 U	*N* = 197: High Hb(65), Low Hb(132)	L: 10.9 vs 10.5 H: 10.8 vs 11.0	No difference in time to dialysis, creatinine clearance, change in creatinine clearance or death.
Pfeffer 2009 [50]	623 sites in 24 countries	Darbepoetin alfa 0.75 mcg/kg (Q2W and switched to QM); to increase Hb from 10.4 to 12.5 g/dL.	48 months; median duration of 29 months	No ESA until Hb dropped below 9 g/dL, 46% received 1 or more doses of ESA	*N* = 4038. High Hb(2012), low Hb(2026)	L:10.4 vs 10.6 H: 10.5 vs 12.5	No difference in the renal composite endpoint
Ritz 2007 [51]	64 centers in 16 countries	Epoetin β (2000 U/week) to a target Hb of 13–15 g/dL.	15 months	Hb target of 10.5–11.5 g/dL. 13/82 patients received ESA	*N* = 172: High Hb(89), Low Hb(83)	L: 11.7 vs 12.1 H: 11.9 vs 13.5	No effect on the rate of decrease in creatinine clearance, change in eGFR or urine protein
Roger 2004 [52]	Australia and New Zealand	Epoetin α 1X/week to increase Hb from 10 to 13 g/dL	24 months	ESA if Hb below 9 g/dL, 8/78 received ESA	*N* = 155: High Hb(75), Low Hb(80)	L: 11.2 vs 11.0 H: 11.2 vs 12.2	No difference eGFR or creatinine clearance at 2 years
Rossert 2006 [53]	93 centers in 22 countries	Epoetin α (25–100 U/kg 1X/week) to a Hb target of 13–15 g/dL. Median dose was 4,514 IU/week	4 months Hb stabilization then 7.4 months maintenance (high Hb) or 8.3 months (low Hb)	Hb target of 11–12 g/day. 65/195 received at least 1 ESA dose. Ave dose 2,730 IU/week (333–7667)	*N* = 390: High Hb(195), Low Hb(195)	L: 11.5 vs 11.7 H: 11.6 vs 13.9	No significant differences in rates of decrease in eGFR
Singh 2006 [54]	130 sites in USA	Epoetin α 1x/week to achieve Hb target of 13.5 g/dL. Ave 11,215 U/week	Median duration 16 months; 661 patients (46.2%) completed 36 months	Target Hb of 11 g/dL (709/717 received ESA) Ave dose 6276 U/week	*N* = 1432: High Hb (715), Low Hb (717)	L: 10.1 vs 11.3 H: 10.1 vs 12.6	No difference in hospitalization for RRT
Villar 2011 [55]	15 centers in France	ESA to target a Hb of 13–14.9 g/dL. Mean weekly ESA dose 6028 ± 6729 IU	24 months	Target Hb of 11–12.9 g/dL. Mean dose 1558 ± 1314 UI/week	*N* = 89: High Hb (46), Low Hb (43)	L: 11.5 vs 11.9 H: 11.4 vs 13.2	No difference in proteinuria or decline in eGFR (2 years)

Patients that progressed to RRT included those that began dialysis or received a transplant. In one trial a patient withdrew because of sepsis and AKI [48]. This event was included in the RRT endpoint of that study. No patients progressed to dialysis in either arm of the Lim 1989 [42] trial making it unsuitable for inclusion in a meta-analysis with a RRT endpoint.

The remaining 18 anemia correction trials had a combined total of 8020 subjects; 3964 in the treatment arm (higher Hb) and 4056 in the comparator (low Hb control) arm. Trials were of varying size; 3 had over 600 subjects. The initial and achieved Hbs in the 2 groups are shown in Table 4.

Overall, 768 (19.4%) of subjects in the treatment arm and 786 (19.3%) in the control arm, progressed to RRT (Fig. 6). With meta-analysis, the RR (random effects) of progression to RRT was 1.04 [0.91, 1.18] with low heterogeneity (I^2^ = 25.0%). This lack of effect on disease progression is supported in 18 trials by other assessments of change in renal function, including proteinuria, or creatinine based markers where there were no significant differences reported between groups (Table 4). However, in one trial time to a doubling in serum creatinine was significantly slower in the ESA group (Kuriyama 1997) [41]. This anemia correction meta-analysis does not assess direct ESA effects per se because subjects in both arms may have received ESAs. However, Hb levels increased in the ESA treatment/high Hb arms. Thus the absence of benefit argues that anemia correction per se is not reno-protective.

In 6 of the 18 anemia correction trials, subjects in the comparator arm did not receive ESAs [18, 19, 38–43]. These trials included a total of 268 subjects. 42 of 129 in the ESA group (33%) and 60 of 139 in the control group (43%) progressed to dialysis. Meta-analysis showed a trend towards improvement in the progression to RRT in the ESA treatment group but this did not reach statistical significance; the RR according to the random effects model was 0.79 [0.6, 1.04] (Fig. 6). The result was similar using the mixed effects model. Heterogeneity was low. Measures of serum creatinine over time showed no statistical difference in 6 of the 7 trials. Thus this select analysis also does not support either direct or indirect (anemia correction) beneficial effect on renal disease progression by ESAs.

## Discussion

We assessed potential beneficial effects of ESA treatment on acute or chronic renal disease. One potential benefit is that ESAs might increase renal tissue survival and therefore renal function following ischemic events due to an interaction of ESAs with receptors resident on the surface of renal cells resulting in an anti-apoptotic effect. Alternatively, there may be mitigation of the negative effects of anemia, since anemia is associated with an increased risk of renal disease progression and allograft loss over the long term [56, 57]. However, these meta-analyses showed no clear benefit of short-term ESAs in AKI and transplant trials, where there was little change in Hb levels, arguing an absence of direct benefit. There was also no significant ESA benefit in longer-term anemia correction trials, regardless of whether the comparator group received or did not receive ESAs. Thus there appeared to be little short or long-term reno-protective benefit of ESAs, via direct (via activation of EPOR or via an interaction of ESA with an EPOR:CD131 hybrid receptor [9]) or indirect (increased Hb) mechanisms.

The lack of clear benefit of ESAs on renal disease is consistent with earlier meta-analyses. A meta-analysis with patients at risk for AKI showed no benefit of ESAs on incidence of AKI [58]. Another meta-analyses of effects of ESAs on CKD patients also showed no clear benefit on progression to RRT, comparing ESA treatment to no treatment [59] or comparing high vs low Hb targets [60, 61], nor was there was an association between ESA dose and annual GFR change or progression to ESRD [62].

Overall and to date, the potential cyto-protective effects of ESAs reported in animal models have generally not translated into benefit in humans, according to other studies examining benefit with other ischemic tissues [63]. There was no significant benefit of ESAs on infarct size in a meta-analyses of patients with acute ST-segment elevation myocardial infarction [64, 65], and no effect on nonfatal heart related events in a meta-analysis of ESA-treated patients with heart failure [66]. There was also no difference in a meta-analysis of retinopathy of prematurity in infants treated with ESAs [67]. There was no benefit of either ESA or increased Hb in an ESA trial on patients with traumatic brain injury [68, 69], and there was no benefit in a phase 3 trial with ESA treatment of stroke patients [70]. Taken together, these observations suggest that ESAs may not have the broad, robust, non-hematopoietic protective abilities described by some investigators, at least not in humans.

The gap between preclinical reports of benefit of ESAs in animals, and the absence of similar robust benefit in humans, has several explanations. Dose and dose regimens may be different, or the animal studies used homogeneous animal types under controlled conditions that cannot be mimicked in the clinic. Another possibility is that a benefit may have been unobservable because of the trial designs used. In this AKI meta-analysis the subjects were primarily cardiac patients and did not have only ischemia to the kidney as in animal studies and therefore may be immune to potential reno-protective ESA benefits.

There could also be other induced mechanisms that may confound the outcome data. For example, sepsis can affect outcomes and blood pressure can increase with ESA treatment and can negatively correlate with renal outcomes [71, 72]. However, control of blood pressure did not affect progression to ESRD in a clinical trial [73].

Alternatively, the beneficial conclusions of preclinical animal studies need to be reconsidered. There are many reports in animals showing a lack of effect of ESAs [1, 74]. The reno-protective hypothesis assumes that EPOR is present, and functional, at significant level on the surface of renal cells. However reports of EPOR presence are either assumed according to responses in tissue culture and in animals, or based on western or immunohistochemistry studies with anti-EPOR antibodies now shown to be nonspecific [75]. Recently a specific antibody to EPOR was discovered and western blots on renal tissue showed few, if any, detectable EPOR raising further questions about the validity of the hypothesis [10].

These meta-analyses have limitations. Majorities of included trials were small, single center, and had modest event rates. The anemia correction trials were larger, but conclusions around direct effects were confounded by the frequent use of ESAs in the comparator arm, though trials where the comparator arm did not receive ESAs similarly showed no benefit. Within each grouping (CKD progression, AKI, transplantation) there were differences in patient selection, treatment regimen and outcome definition. Finally, the meta-analyses were based on aggregated, not individual patient level data, which precluded adjustments for confounding factors such as age and comorbidities.

## Conclusions

In contrast to some preclinical studies demonstrating reno-protection by ESAs in animals, anemia correction, prophylaxis or post-injury intervention with ESAs provided no significant clinical reno-protection in humans. This suggests that ESAs may not have robust, nor reproducible direct, or indirect, benefits on renal function.

## Abbreviations

AKI: Acute kidney injury
AKIN: Acute kidney injury network
CABG: Coronary artery bypass grafting
CKD: Chronic kidney disease
DGF: Delayed graft function
eGFR: Estimated glomerular filtration rate
EPO: Erythropoietin
EPOR: EPO receptor
ESA: Erythropoiesis stimulating agent
Hb: Hemoglobin
ITT: Intention to treat
RCT: Randomized controlled trial
RR: Risk ratios
RRT: Renal replacement therapy
SCr: Serum creatinine
